# Contemporary snapshot of tumor regression grade (TRG) distribution in locally advanced rectal cancer: a cross sectional multicentric experience

**DOI:** 10.1007/s13304-021-01044-0

**Published:** 2021-04-05

**Authors:** Paola Germani, Francesca Di Candido, Daniel Léonard, Dajana Cuicchi, Ugo Elmore, Marco Ettore Allaix, Vittoria Pia Barbieri, Laura D’Allens, Seraina Faes, Marika Milani, Damiano Caputo, Carmen Martinez, Jan Grosek, Valerio Caracino, Niki Christou, Sapho X. Roodbeen, Umberto Bracale, Aurelia Wildeboer, Antonella Usai, Michele Benedetti, Alessandro Balani, Giuseppe Piccinni, Marco Catarci, Paolo Millo, Nicole Bouvy, Francesco Corcione, Roel Hompes, Frédéric Ris, Massimo Basti, Ales Tomazic, Eduardo Targarona, Alessandro Coppola, Andrea Pietrabissa, Dieter Hahnloser, Michel Adamina, Massimo Viola, Mario Morino, Riccardo Rosati, Gilberto Poggioli, Alex Kartheuser, Antonino Spinelli, Nicolò de Manzini, the TRG Snapshot Study Group, Gabriele Bellio, Cristiana Iacuzzo, Annalisa Zucca, Pio Corleone, Fabiola Giudici, Silvia Palmisano, Michele Carvello, Christophe Remue, Radu Bachmann, Nicolas Lombard, Christine Pirlet, Andries Ryckx, Simonetta Massaron, Luigi Pugliese, Roberto Coppola, Cecilia Ferrari, Simone Castiglioni, Elisa Ponte, Serena Concina, Arthur Piveteau, Yongbo An, Emanuela Cagnazzo, Marina Troian

**Affiliations:** 1 Department of Medical, Surgical and Health Sciences, University of Trieste, Strada di Fiume 447, Trieste, Italy; 2 Department of General Surgery, Academic Hospital of Trieste, Strada di Fiume 447, Trieste, Italy; 3 Colon and Rectal Surgery Unit, Humanitas Research Hospital, Milano, Italy; 4 Colorectal Surgery Unit, Saint-Luc University Hospital, Université Catholique de Louvain (UCL), Brussels, Belgium; 5 Department of Medical and Surgical Sciences, Surgery of the Alimentary Tract, Sant’Orsola Hospital, Alma Mater Studiorum University of Bologna, Bologna, Italy; 6 Department of Gastrointestinal Surgery, San Raffaele Scientific Institute, Vita e Salute University, Milano, Italy; 7 Department of Surgical Sciences, University of Torino, Torino, Italy; 8 Department of General Surgery, Cardinale Panico di Tricase Hospital, Lecce, Italy; 9 Department of Surgery, Cantonal Hospital Winterthur, Winterthur, Switzerland; 10 Department of Visceral Surgery, Centre Hospitalier Universitaire Vaudois Lausanne, Lausanne University Hospital, Lausanne, Switzerland; 11 Department of Surgery, Fondazione IRCCS Policlinico San Matteo, Pavia, Italy; 12 Department of General Surgery, University Campus-Biomedico Roma, Roma, Italy; 13 Department of Medicine of the Autonomous University of Barcelona, Hospital de la Santa Creu i Sant Pau, Barcelona, Spain; 14 Department of Abdominal Surgery, University Medical Centre Ljubljana, Ljubljana, Slovenia; 15 Department of General Surgery UO III, Ospedale Santo Spirito, Pescara, Italy; 16 Division of Digestive Surgery, University Hospitals of Geneva, Genève, 1211 Switzerland; 17 Department of Surgery, Amsterdam UMC, University of Amsterdam, Amsterdam, The Netherlands; 18 Department of Advanced Biomedical Sciences, Federico II University, Naples, Italy; 19 Department of Surgery, Maastricht University Medical Centre, Maastricht, The Netherlands; 20 Department of Surgery, General and Urgent Surgery Unit, Regional Hospital “U. Parini”, Aosta, Italy; 21 General Surgery Unit, “C. E G. Mazzoni” Hospital, Ascoli Piceno, Italy; 22 Department of General Surgery, Santa Maria Hospital GVM Care and Research, Bari, Italy; 23 Department of Surgery, San Polo Hospital, Monfalcone, Italy; 24 Department of Public Health, Federico II University, Naples, Italy; 25 Division of Radiology, University Hospitals of Geneva, Genève, 1211 Switzerland

**Keywords:** Rectal cancer, Pathologic complete response, Neoadjuvant therapy, Tumor regression grade

## Abstract

Pre-operative chemoradiotherapy (CRT) followed by surgical resection is still the standard treatment for locally advanced low rectal cancer. Nowadays new strategies are emerging to treat patients with a complete response to pre-operative treatment, rendering the optimal management still controversial and under debate. The primary aim of this study was to obtain a snapshot of tumor regression grade (TRG) distribution after standard CRT. Second, we aimed to identify a correlation between clinical tumor stage (cT) and TRG, and to define the accuracy of magnetic resonance imaging (MRI) in the restaging setting. Between January 2017 and June 2019, a cross sectional multicentric study was performed in 22 referral centers of colon-rectal surgery including all patients with cT3-4Nx/cTxN1-2 rectal cancer who underwent pre-operative CRT. Shapiro–Wilk test was used for continuous data. Categorical variables were compared with Chi-squared test or Fisher’s exact test, where appropriate. Accuracy of restaging MRI in the identification of pathologic complete response (pCR) was determined evaluating the correspondence with the histopathological examination of surgical specimens.

In the present study, 689 patients were enrolled. Complete tumor regression rate was 16.9%. The “watch and wait” strategy was applied in 4.3% of TRG4 patients. A clinical correlation between more advanced tumors and moderate to absent tumor regression was found (*p* = 0.03). Post-neoadjuvant MRI had low sensibility (55%) and high specificity (83%) with accuracy of 82.8% in identifying TRG4 and pCR.

Our data provided a contemporary description of the effects of pre-operative CRT on a large pool of locally advanced low rectal cancer patients treated in different colon-rectal surgical centers.

## Introduction

Colorectal cancer represents the third most common cancer and the second cause of cancer-related mortality worldwide [1]. In about 35% of cases, patients present with rectal cancer [2] and, in case of locally advanced low rectal lesions, pre-operative chemoradiotherapy (CRT) followed by surgical resection with total mesorectal excision is currently the standard treatment, reducing local recurrence from 25% to 5–10% and significantly improving overall survival (OS) [3–9].

Response to neoadjuvant CRT is evaluated on the surgical specimen by assessing the stage and tumor regression grade (TRG). Among the many TRG systems which aim to categorize the amount of regressive changes after CRT, the Dworak classification is one of the most commonly used for rectal cancer [10–12]. Response rate to pre-operative CRT can be quite variable in the literature [10–12], with pathological complete response (pCR) reported in 8–21% of patients and partial responses reported in about 40% of patients [4, 13–15]. Correct assessment of TRG, as well as thorough evaluation of the tumor and nodal status (and, in case, of the metastatic status) on the surgical specimen (i.e. ypTNM) [16], are of paramount importance to predict prognosis as long-term oncological outcomes are significantly better in patients with complete regression compared to those with partial or absent regression [3, 4, 17–20].

According to these considerations, in 2004 Habr-Gama proposed the “watch and wait” strategy for patients with clinical complete response (cCR), demonstrating that it was possible to avoid surgical resection and its related morbidity and long-term sequelae without affecting OS and disease-free survival (DFS) [21]. Nevertheless, more recent studies have failed to reach the same results [22]. The main challenge remains the correct identification of patients eligible for this approach. In fact, cCR does not always correspond to pCR, thus determining an increased risk of tumor regrowth in poorly selected patients [18, 20, 23], although salvage surgery can still be performed without reduction in survival [17, 18]. Consequently, current ESMO guidelines endorse a “watch and wait” strategy only for cCR cases that are poor surgical candidates, recommending at any rate that patients should be warned about the slightly increased risk of pelvic relapse and distant metastases [2]. Another topic of debate is the fact that patients undergoing a “watch and wait” strategy demand a strict follow-up protocol, but no agreement has been reached on the timing and type of evaluations (i.e. clinical, endoscopic, and radiological) required [24].

As a matter of fact, the therapeutic approach to rectal cancer is constantly changing and defining the best treatment for each patient is currently one of the biggest challenges in clinical oncology. The primary aim of this study was to obtain a snapshot of TRG distribution after standard CRT in some European referral centers of colorectal surgery. Second, we aimed to identify a correlation between the clinical stage of the tumor (cT) and TRG, and to define the accuracy of magnetic resonance imaging (MRI) in the restaging setting. Finally, we aimed to present the current attitudes of colorectal surgeons towards cCR patients (i.e. surgery vs. conservative treatment).

## Methods

### Study design

A cross sectional multicentric study was performed in 14 Italian and 8 European referral centers of colorectal surgery between January 2017 and June 2019. All the clinical and pathological data were drawn together in a single anonymous database. The manuscript adheres to the Strengthening the Reporting of Observational Studies in Epidemiology (STROBE) Statement [25].

Inclusion criteria were: locally advanced rectal carcinoma (cT3–cT4 and/or cN1–cN2 at diagnosis); neoadjuvant long-course CRT; surgical resection with curative intent or “watch and wait” strategy with either full-thickness or endoscopic biopsy. Patients were excluded in case of: stage IV at diagnosis; neoadjuvant short-course radiotherapy (RT); neoadjuvant chemotherapy (CT) without RT; lack of significant data (i.e. pre-operative c-stage and/or yc-stage, histology, TRG, type of pre-operative regimen).

After being diagnosed with rectal cancer, all patients underwent a staging MRI. Primary tumor (T) and nodal involvement (N) were registered according to the American Joint Committee on Cancer (AJCC), 7th edition [16]. Infiltration of the mesorectal fascia, and distance between primary tumor and mesorectal fascia were recorded. The mesorectal fascia was considered infiltrated in case of tumor distance ≤ 5 mm and non-infiltrated in case of tumor distance ≥ 6 mm. The same parameters were recorded in the restaging setting, where available.

Different long-course CRT regimens were reported. After pre-operative treatment, patients underwent surgical resection with curative intent. The “watch and wait” strategy was considered an option for patients with cCR, according to either surgeon’s or patient’s preference. Retrospective analysis of different surgeon’s attitudes was performed.

All surgical specimens were classified according to the AJCC staging system, 7th edition [16]. Data about regressive changes after CRT, infiltration of the mesorectal fascia, and distance between primary tumor and mesorectal fascia were recorded for all patients. Tumor regression grading was evaluated according to the Dworak classification [10].

### Statistical analysis

Continuous data were summarized by median and range (minimum–maximum) and were tested for normality with the Shapiro–Wilk test. Post-hoc tests and pairwise comparisons through Mann–Whitney test were conducted and *p* value adjusted using Holm method. Categorical variables were reported as absolute and relative frequencies (percentages) and compared with Chi-Squared test or Fisher’s exact test where appropriate.

The capability of restaging MRI at correctly identifying pCR patients was evaluated with sensibility, specificity, and accuracy. True positive results were considered in case of ycT0 patients at restaging MRI corresponding to TRG 4 specimens on histopathological examination.

Statistical analysis was conducted with R version 3.5.0 and STATA 14.2 (StataCorp, College Station, TX, USA). All *p* values (*p*) were two-tailed and differences were considered statistically significant when *p* < 0.05.

## Results

**Fig. 1:**
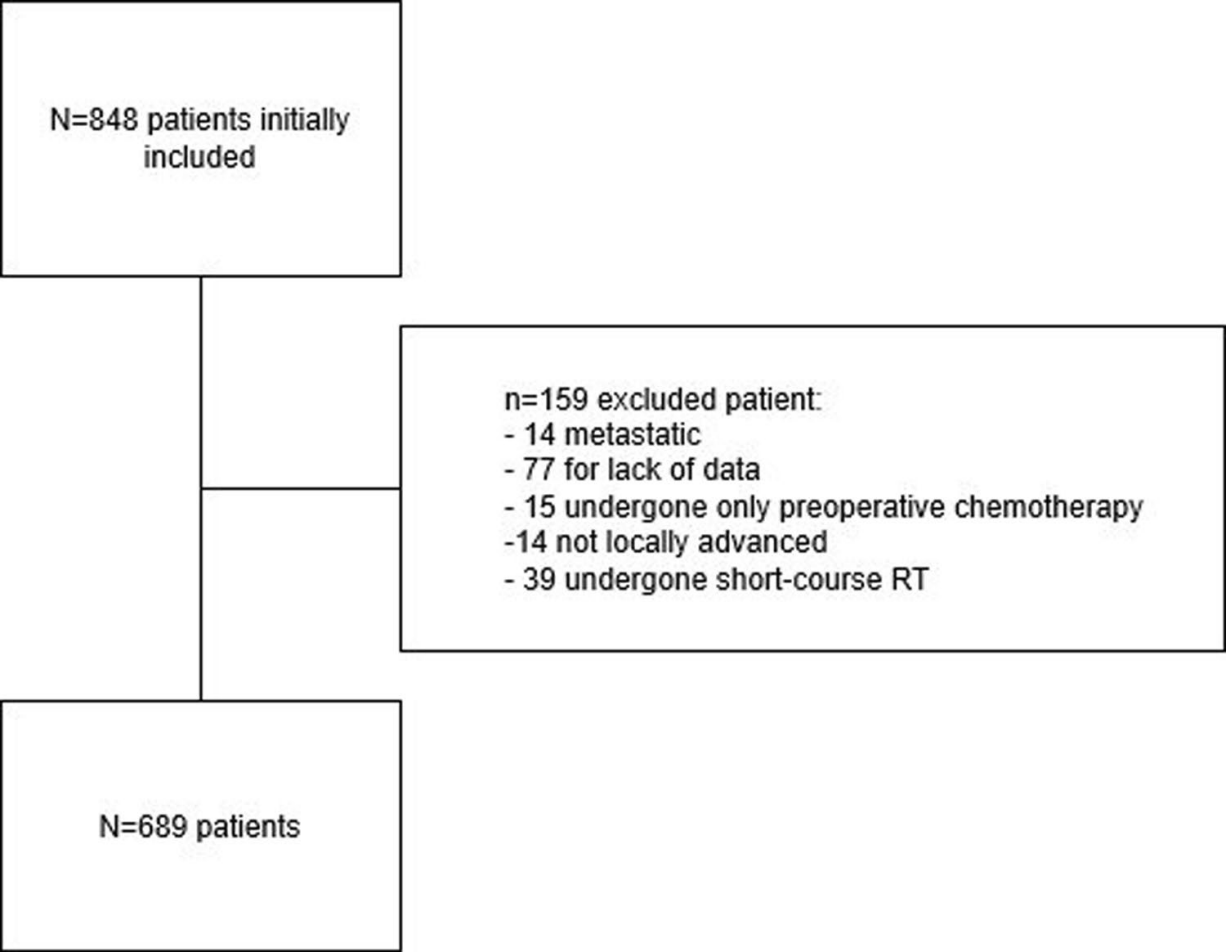
Patients selection

A total of 848 patients from 22 European referral centers of colorectal surgery were initially considered for the present study. Of these, 159 were excluded for the following reasons: 14 were metastatic, 14 were not locally advanced, 15 underwent only neoadjuvant chemotherapy (CT), 39 underwent short-course radiotherapy (RT), and 77 were missing significant data (i.e. pre-operative c-Stage and/or yc-Stage, histology, TRG, type of pre-operative regimen). Overall, 689 patients were included for final analysis (Fig. 1). Population distribution and type of long-course CRT regimens are summarized in Tables 1 and 2.

**Table 1:** Population distribution

Centre	Total number (%)
Italy (14 centres)	442 (64.2%)
Switzerland (3 centres)	89 (12.9%)
Belgium (1 center)	72 (10.4%)
Netherlands (2 centres)	39 (5.7%)
Spain (1 center)	25 (3.6%)
Slovenia (1 center)	22 (3.2%)
Total (22 center)	689

**Table 2:** Pre-operative CRT regimens

Chemotherapic agent	No. (%)
Capecitabine	474 (68.8%)
Folfox	32 (4.6%)
5-FU	30 (4.3%)
Folfiri + panitumumab	2 (0.3%)
Folfox + bevacizumab	2 (0.3%)
Folfoxiri	2 (0.3%)
Oxaliplatino	2 (0.3%)
5-FU + levofolinile	1 (0.15%)
Docetaxel	1 (0.15%)
Folfiri	1 (0.15%)
Folfirinox	1 (0.15%)
Atezolizumab	1 (0.15%)
Capox + bevacizumab	1 (0.15%)
Missing data	139 (20.2%)

### TRG distribution

**Fig. 2:**
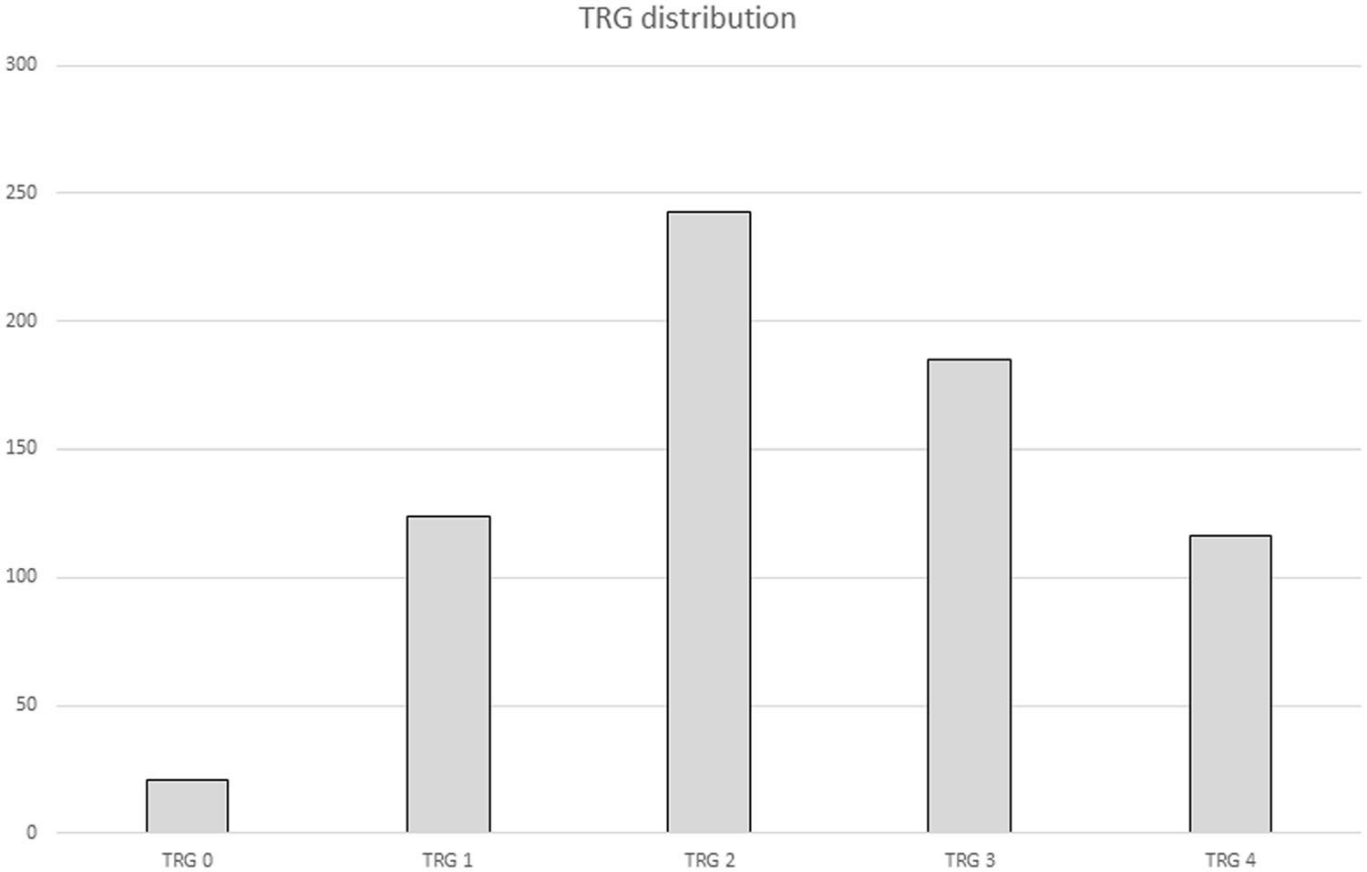
TRG distribution

Overall, TRG 0 was recorded in 21 (3%) patients, TRG 1 in 124 (18%) patients, TRG 2 in 243 (35.3%) patients, TRG 3 in 185 (26.8%) patients, and TRG 4 in 116 (16.9%) patients. Results are displayed in Fig. 2.

### Surgeon’s attitude in case of TRG 4 patients

A retrospective analysis of the therapeutic strategy in case of TRG 4 patients was performed. Overall, 111 (95.7%) patients underwent surgical resection and 5 (4.3%) patients underwent full-thickness biopsy as part of a “watch and wait” strategy. The reason behind the choice was reported in 42 cases and it was predominantly made by the surgeon (i.e. surgeon’s choice in 40 cases vs. patient’s choice in two cases).

Among TRG 4 patients, despite complete tumor response (ypT0), a non-complete nodal response was observed in 11 (9.9%) cases on surgical specimen.

### Association between TRG and clinical T-stage (cT)

Considering the clinical stage of the tumor at diagnosis (i.e. cT), more advanced lesions were less likely to achieve complete regression after neoadjuvant CRT. Indeed, TRG 4 rate was 29.0% for cT1–cT2 tumors, 17.2% for cT3 tumors, and 11.6% for cT4 tumors, respectively (*p* = 0.09). Near-complete (i.e. TRG 3) and complete (i.e. TRG 4) regression significantly correlated with tumor size, as it was reported in 58.1% of cT1–cT2 tumors, 45.6% of cT3 tumors, and 30.4% of cT4 tumors (*p* = 0.003).

Data on the distance between the tumor and the mesorectal fascia were available for 538 (78.1%) patients. Tumors close to or infiltrating the mesorectal fascia (≤ 5 mm) were less likely to achieve TRG 4 response (*p* = 0.005).

**Fig. 3:**
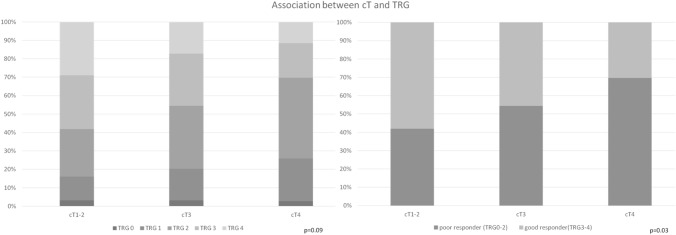
Association between cT and TRG

**Fig. 4:**
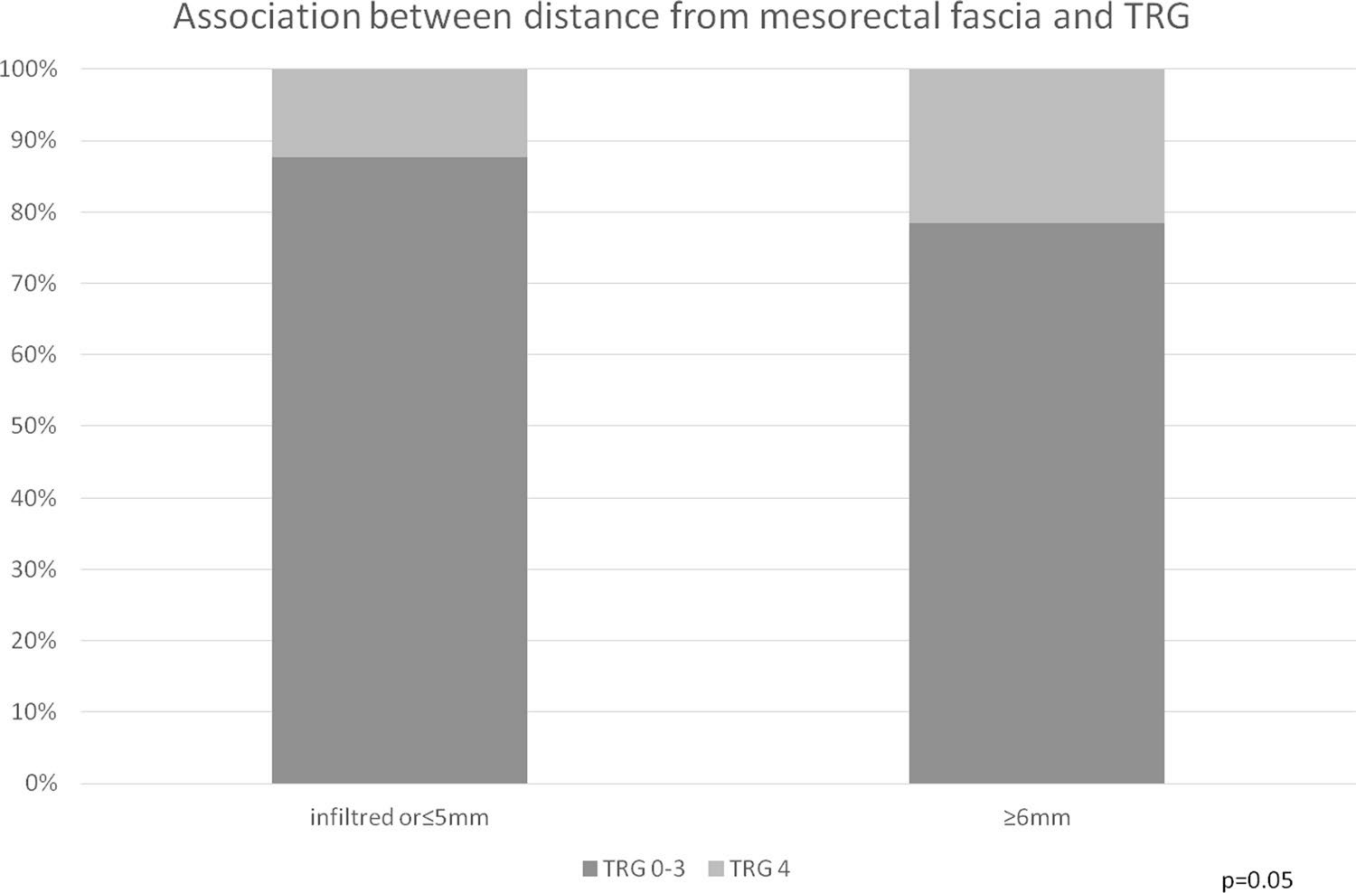
Association between distance from mesorectal fascia and TRG

Results are summarized in Figs. 3 and 4 and Tables 3 and 4.

**Table 3:** Association between cT and TRG

	TRG 0	TRG 1	TRG 2	TRG 3	TRG 4	Tot	
cT1-2	1 (3.2%)	4 (12.9%)	8 (25.8%)	9 (29.05%)	9 (29.05%)	31	*p* = 0.09
cT3	17 (3,1%)	94 (17.2%)	186 (34.1%)	155 (28.4%)	94 (17.2%)	546	
cT4	3 (2.7%)	26 (23.2%)	49 (43.8%)	21 (18.7%)	13 (11.6%)	112	
Tot	21	124	243	185	116	689	

	Poor responder (TRG0–2)	Good responder (TRG3–4)	Tot	
cT1–2	13 (41.9%)	18 (58.1%)	31	*p* = 0.003
cT3	297 (54.4%%)	249 (45.6%)	546	
cT4	78 (69.6%)	34 (30.4%)	112	
Tot	388	301	689

**Table 4:** Association between distance from mesorectal fascia and TRG

	TRG 0–3	TRG 4	Total	
Infiltrated or ≤ 5 mm	317 (87.6%)	45 (12.4%)	362	*p* = 0.005
≥ 6 mm	138 (78.4%)	38 (21.6%)	176	
Total	455	83	538	

### Accuracy of restaging MRI

Restaging MRI was performed in 607 (88.1%) patients. In 82 (11.9%) patients, it was omitted according to center protocol. All patients, in whom restaging MRI was omitted according to center protocol, underwent surgical resection, including TRG4 patients (i.e. 10 cases).

Overall, MRI showed a 55% sensibility, 83% specificity, and 82.8% accuracy for the correct identification of TRG 4 patients (*p* < 0.001). When analyzing the nodal status, MRI showed similar results, with a 52% sensibility, 85% specificity, and 84.5% accuracy for correct identification of lymph node involvement (*p* < 0.001).

Generally, MRI correctly identify ypT and ypN stages in 49.8 and 66.2% of cases, respectively. Overestimation occurred in 41.3% of patients for T stage and 25.4% of patients for N stage, respectively. Underestimation occurred in 8.9% of patients for T stage and in 8.4% of patients for N stage, respectively.

## Discussion

The present paper evaluated the factors associated with tumor regression, analyzed the accuracy of restaging MRI, and described real-life therapeutic strategies in case of complete response to CRT among a sample of Italian and European surgeons.

Overall, our results are consistent with literature data. Specifically, TRG 4 was recorded in 16.9% of patients. Of these, the majority underwent surgical resection; whereas, a “watch and wait” strategy was performed only in 4.3% of cases. This could be due to lack of standardized protocols, but a major concern is certainly represented by the fact that complete tumor regression does not always correspond to complete response in the lymph nodes. In the present series, 9.9% of TRG 4 patients presented residual nodal disease (i.e. ypN+) at histological examination of the surgical specimen. This is consistent with literature data, reporting that up to 10% of ypT0 patients still present metastatic lymph nodes at the time of surgery, thus determining an increased risk of local recurrence and lower 5-year DFS and OS rates [20, 23, 26, 28].

Tumor regression grade was also found to significantly correlate with clinical T stage and distance of the tumor from the mesorectal fascia. According to our results, early clinical T stages and tumors with a distance ≥ 6 mm from the mesorectal fascia were more likely to achieve complete tumor regression (i.e. TRG 4). This is in accordance with the literature. Several studies have analyzed pCR predictive factors and clinical T stage has been widely recognized as a strong predictive factor of tumor response. Tan et al. [27] have recently reported that only 12% of cT4 patients achieve pCR compared to 21% of cT3, 23% of cT3, and 27% of cT1 patients, respectively (*p* < 0.001). Therefore, neoadjuvant treatment may play a role also in early rectal cancers, and specifically cT2N0 lesions. Recent studies comparing surgery alone versus CRT and local excision for cT2N0 patients showed similar oncologic results [28–30]; however, further studies with longer follow-up periods and larger series are required to draw definitive conclusions.

A tailored therapeutic approach strongly relies on accurate pre-operative imaging and MRI should be highly effective in predicting the pathologic state of rectal cancer patients undergoing neoadjuvant treatment. In this study, restaging MRI showed a low sensitivity (55%) but high specificity (83%) in the identification of ypT0 tumors, with an accuracy of 82.8%. Similar results were obtained for the nodal status and overall, restaging MRI correctly identified 49.8% of T stages and 66.2% of N stages, respectively. As reported by other studies, stage overestimation was more common than stage underestimation. In 2014, Lee et al. [31] analyzed 150 patients with locally advanced rectal cancer undergoing post-CRT MRI and observed that pathologic T stage-matched restaging MRI findings in 64.7% of patients, with 24.0% of cases being over-staged. Similarly, pathologic N stage-matched restaging MRI findings in 56.6% of patients, with 36.0% of cases being over-staged. The authors concluded that restaging MRI has low accuracy for the prediction of pathologic T and N classifications in patients-receiving pre-operative CRT. More recently, Cho et al. [34] reported that magnetic resonance TRG (mrTRG) has a sensibility of 37.9%, a specificity of 76.5%, and an accuracy of 66.3%, in the identification of ypT0 tumors. Furthermore, Sclafani et al. [32] reported a low agreement between mrTRG and pTRG, although sensibility and specificity were high (74.4 and 62.8%, respectively).

MRI accuracy in the assessment of TRG can be increased by diffusion-weighted imaging (DWI). However, over-staging and under-staging remains a problem, as the main limitation of MRI lies in the difficulty at discriminating between residual tumor, fibrosis, edema, and inflammation. Moreover, small residual cells may not be seen on radiological evaluation [33, 34]. Some authors believe that the addition of endoscopic ultrasound (EUS) imaging to MRI might increase the accuracy of post-treatment staging; however, a satisfactory agreement has yet to be reached [33, 35]. In this setting, radiomic seems to be a promising technique. Preliminary results, recently published by the MSKCC group, showed a better performance of radiomic in the identification of a complete response compared to T2-weighted MRI and DWI sequences [35], thus representing a significant potential guidance in the individualization of the most appropriate therapeutic approach for each patient [35–37].

The present study had several limitations. First, it was retrospective and not fully representative of European surgical attitudes in rectal cancers, although several referral centers were included in the analysis. Second, the study evaluated only some predictive factors of complete tumor response, such as clinical stage, neoadjuvant therapy, and distance of the tumor from the mesorectal fascia. However, other factors, including molecular pathways and tumoral markers, may play a role as well. Third, the population was not homogeneous and included patients who underwent different schemes of chemotherapy, thus potentially influencing outcomes.

Nevertheless, this is one of the first studies to offer some sort of contemporary description of the effects of pre-operative CRT on a large pool of locally advanced low rectal cancer patients treated in different Italian and European colorectal surgical centers. Moreover, despite being retrospective, the relatively large sample size collected in a short and recent period allowed to obtain clinically significant results and all data were drawn out from primary referral colorectal centers. Therefore, we believe that the results of this study could be useful for the definition of an ever more tailored approach and the analysis of current critical issues could promote further prospective multicentric evaluations on multimodal therapy for rectal cancer.

## Data Availability

The data will not be deposited.

## Code availability

Not aplicable.
